# Single-Molecule Techniques to Study Chromatin

**DOI:** 10.3389/fcell.2021.699771

**Published:** 2021-07-05

**Authors:** Anna Chanou, Stephan Hamperl

**Affiliations:** Chromosome Dynamics and Genome Stability, Institute of Epigenetics and Stem Cells, Helmholtz Zentrum München, Munich, Germany

**Keywords:** single-molecule techniques, chromatin accessibility, electron microscopy, third-generation sequencing, histone modifications, magnetic/optical tweezers, chromatin replication/transcription, DNA fiber analysis

## Abstract

Besides the basic organization in nucleosome core particles (NCPs), eukaryotic chromatin is further packed through interactions with numerous protein complexes including transcription factors, chromatin remodeling and modifying enzymes. This nucleoprotein complex provides the template for many important biological processes, such as DNA replication, transcription, and DNA repair. Thus, to understand the molecular basis of these DNA transactions, it is critical to define individual changes of the chromatin structure at precise genomic regions where these machineries assemble and drive biological reactions. Single-molecule approaches provide the only possible solution to overcome the heterogenous nature of chromatin and monitor the behavior of individual chromatin transactions in real-time. In this review, we will give an overview of currently available single-molecule methods to obtain mechanistic insights into nucleosome positioning, histone modifications and DNA replication and transcription analysis—previously unattainable with population-based assays.

## Introduction

The remarkable length and complexity of eukaryotic genomes poses several challenges to the cell. The limited 3D space in the nucleus requires a high degree of DNA compaction, while maintaining sufficient accessibility for gene expression. In order to overcome this challenge, cells package the genome into a nucleoprotein complex termed chromatin (Igo-Kemenes et al., 1982). Chromatin plays a central role for all DNA-dependent transactions, such as replication, transcription, recombination, and DNA repair, thus requiring tight regulation (Kornberg and Lorch, 2020).

The nucleosome is the basic unit of chromatin, each occupying 147 bp of DNA wrapped around histone octamers. Each octamer consists of two histone H3-H4 dimers that complexes with two histone H2A-H2B dimers (McGhee and Felsenfeld, 1980; Luger et al., 1997). Individual nucleosome core particles (NCPs) are separated by short 15–50 bp of linker DNA, giving chromatin the typical appearance of “beads on a string,” as shown by classical electron microscopy studies (Olins and Olins, 1978). In general, the tight interaction of DNA and octamers is inhibitory to most nuclear processes and thus, nucleosomes must be dynamically repositioned to allow binding or repel effector proteins from specific regulatory DNA regions. Apart from intrinsically favorable or unfavorable DNA sequences for nucleosome formation (Travers et al., 2010), other cellular components, such as transcription factors and ATP-dependent chromatin remodeling machines contribute to the chromatin landscape *in vivo* (Dou and Gorovsky, 2000; Jenuwein, 2001; Lusser and Kadonaga, 2003; Heintzman et al., 2009; Bell et al., 2011). In addition, the unstructured N-terminal tails of histones protrude from the nucleosomal core, presenting platforms of discrete epitopes that can be targeted for addition or removal of distinct post-translational modifications (PTMs). More than a hundred histone modifications are described including the acetylation of lysines, the methylation of arginines and lysines, and the phosphorylation of serines and threonines (Tyler et al., 1999; Strahl and Allis, 2000; Jenuwein, 2001; Lusser and Kadonaga, 2003; Gibney and Nolan, 2010). Combining these diverse PTMs in a combinatorial way greatly increases the heterogeneity of distinct chromatin states that can co-exist in a given genome. Broadly, two major categories of chromatin can be distinguished: highly condensed heterochromatin that is transcriptionally inert and more open, transcriptionally active euchromatin.

It is clear that the dynamics, structure and composition of chromatin have a major influence on the transcriptional and replicative output of a genome. To understand these complex processes in more mechanistic details, it is critical to obtain a comprehensive overview of the composition and histone PTM patterns in correlation with the functional state of a locus of interest. Various population-based techniques have been developed to investigate the architecture of chromatin. The genome can be surveyed for exposed regions accessible for nuclease (DNAse-seq, MNase-seq, FAIRE-seq) (Schones et al., 2008; Bianco et al., 2015; Ishii et al., 2015) or transposase attack (ATAC-seq) to distinguish between open regulatory regions of chromatin vs. those protected by nucleosomes (Buenrostro et al., 2015).

To probe for more specific protein-DNA interactions, chromatin immunoprecipitation (ChIP) has been a powerful tool to determine genome-wide binding profiles of transcription factors, histone PTMs or variants and other chromatin-bound factors on the one-dimensional genetic sequence (Schmid et al., 2004; Wal and Pugh, 2012). In recent years, the Cleavage Under Targets & Release Using Nuclease (CUT&RUN) method has emerged as a powerful alternative to ChIP, as the antibody-mediated tethering of MNase releases directly DNA-protein complexes into the supernatant *in situ* without the need for fragmentation and solubilization of total chromatin, which represents a major drawback of ChIP (Meers et al., 2019). However, the binding profiles generated by both methods oversimplify the activities of regulatory elements like enhancers, silencers, insulators, and boundaries, that can affect their corresponding target genes over megabase distances by 2D and 3D chromatin looping (Bonev and Cavalli, 2016). Thus, to characterize the global contacts in 3D physical space, several Hi-C chromosome conformation capture protocols have been developed providing detailed interaction maps up to kilobase pair resolution (Rao et al., 2014). More recently, Micro-C, where MNase is used for fragmentation prior to re-ligation, was shown to provide interaction maps at nucleosome resolution in yeast and several human and murine cell lines (Hsieh et al., 2016).

Despite their usefulness and great insights into the chromatin structure and spatial organization of chromosomes, it is important to realize that all of these genomic methods describe the properties of enormous numbers of molecules, averaging the measured parameters over a large population of molecules and cells. Thus, the behavior of individual molecules with distinct conformations and properties cannot be observed over time. In addition, bulk methods fail to detect rare events that occur only in a small subpopulation of molecules. Therefore, single-molecule methods provide the only possible solution to detect the functional differences and uncover intermolecular heterogeneities, particularly important in the context of chromatin. In this review, we will give an overview of currently available single-molecule methods that provide mechanistic insight into chromatin structure and processes, such as chromatin accessibility, histone modifications, replication and transcription (Supplementary Table 1). For each technique, we start with a brief description of the general principles and provide selected examples how these methods have contributed to our understanding of chromatin which would not have been possible by using bulk and ensemble studies.

## Single-Molecule Approaches to Detect Nucleosome Occupancy and Positioning

### Electron Microscopy After Psoralen Crosslinking

**FIGURE 1:**
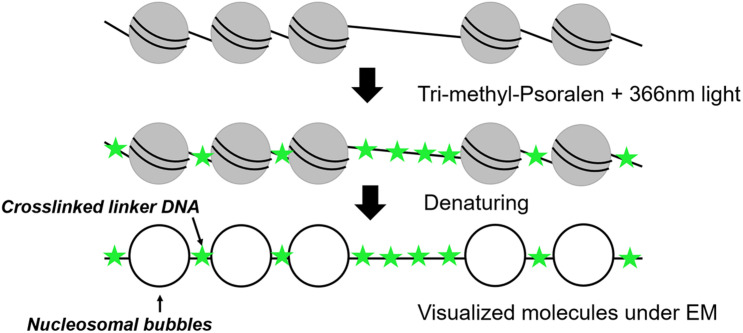
Schematic representation of psoralen crosslinking on a nucleosomal template.

Nucleosomal arrays were first detected by electron microscopy (EM) in 1974 (Olins and Olins, 1974) revealing a “beads on a string” structure (Kornberg, 1974). In 1976, photochemical crosslinking of DNA with derivates of psoralen was used for nucleosome visualization under EM (Hanson et al., 1976; Sogo et al., 1984). Psoralen is a furanocoumarin compound with the ability to intercalate into double-stranded DNA. After irradiation with ultraviolet (UV)-light at 366 nm, psoralen creates covalent crosslinks between pyrimidines of opposite DNA stands (Cimino et al., 1985). The crosslinking occurs in linker DNA, whereas the nucleosomal DNA is protected, which allows to distinguish whether a DNA region had been occupied by a nucleosome or not (Figure 1). After deproteinization, the crosslinked DNA shows a characteristic shift in native agarose gel electrophoresis and can be analyzed by Southern blot analysis (Cimino et al., 1985; Wellinger and Sogo, 1998; Lucchini, 2001). Such experiments could for example demonstrate the co-existence of two major populations of ribosomal DNA (rDNA) genes, a nucleosome-free open state and a nucleosomal closed state (Wittner et al., 2011). However, this bulk analysis could only determine qualitative differences and not inform on the individual nucleosome configurations and heterogeneity among individual molecules.

Thus, the combination of psoralen-crosslinking and visualization by EM can be considered as one of the pioneering tools to determine nucleosome positioning at the single-molecule level. After denaturation of the DNA strands, linker DNA is observed double-stranded, whereas nucleosomal DNA appears as single-stranded DNA bubbles (Brown and Boeger, 2014; Figure 1). Inspection of yeast rDNA chromatin by this technique clearly demonstrated the co-existence of the two classes of rRNA genes at the single-molecule level (Dammann et al., 1993). More recently, this technique was applied to single-gene molecules encompassing the PHO5 promoter in yeast under inducing and non-inducing conditions. Interestingly, both the induced as well as non-induced PHO5 promoter displayed with different frequencies every single combinatorial possibility to occupy the three PHO5 promoter nucleosome positions (2^3^ = 8). This finding suggests that individual PHO5 promoters pass, in a certain sequence, through alternative nucleosome configurations (Brown et al., 2013). Such a large heterogeneity in promoter nucleosome configuration—uncovered by a single-molecule technique—can thus explain gene expression fluctuations and suggest a structural basis for transcriptional bursting. Recently, integration of these single-molecule with other existing data on chromatin accessibility and histone turnover revealed that only few models that include directional sliding and a regulated assembly instead of disassembly process was compatible with the experimental data and suggests PHO5 promoter chromatin opening by binding competition (Wolff et al., 2021). Psoralen crosslinking has also been used to determine the nucleosome positioning at 5S rDNA molecules. The molecules were clustered into 12 different groups exhibiting high level of heterogeneity among the cell population, but the number of molecules with nucleosome-free 5S sequences could recapitulate the expected accessibility and transcriptional activity of 5S rRNA genes (Hamperl et al., 2014). One drawback of this method is the need for special equipment and the costly and time-consuming experimental setup limiting this application to more specialized labs. However, EM remains the only available tool offering sufficient resolution to directly visualize individual nucleosomal footprints at the single-molecule level.

### Methylation Footprinting

**FIGURE 2:**
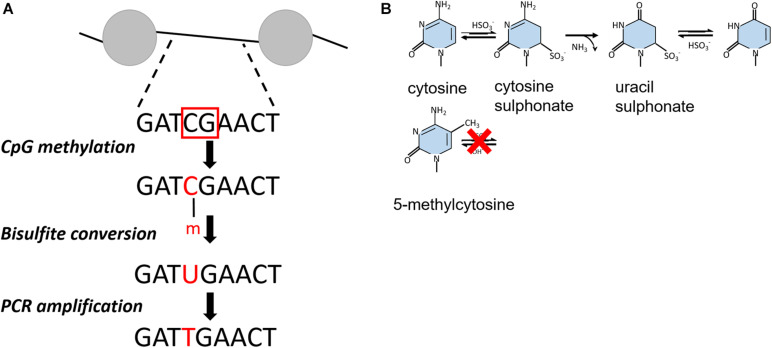
**(A)** Outline of bisulfite conversion on an example DNA fragment. The red squares indicate non-nucleosomal regions recognized by CpG methyltranferases. After bisulfite conversion, cytosines are presented as uracil, which are converted into thymidine. Nucleosomal regions are discriminated from non-nucleosomal by comparing the resulting PCR product with the initial DNA sequence. **(B)** Chemical reaction of bisulfite-catalyzed conversion of cytosine to uracil.

Similar to psoralen only intercalating into accessible, nucleosome-free regions of chromatin, bulk chromatin can be treated with specific DNA methyltransferases that target unprotected cytosines of CpG dinucleotides within free DNA but not the DNA in complex with a nucleosome or a transcription factor (Kladde and Simpson, 1994; Miranda et al., 2010). Upon bisulfite conversion, unmethylated cytosine residues are deaminated to uracil but methylated cytosine (5-mC) residues remain inert to this reaction (Figures 2A,B). The methylated DNA fragment is amplified by PCR and cloned into a vector for amplification in *E. coli*. By comparison to a reference or untreated DNA sequence, methylation events can therefore be detected directly by traditional Sanger or Illumina sequencing, providing a single-molecule readout with base-pair resolution that is highly quantitative (Miranda et al., 2010; Li and Tollefsbol, 2011).

Unlike yeast and Drosophila, mammalian genomes show endogenous DNA methylation at CpG dinucleotides (Stadler et al., 2011), preventing the use of exogenous CpG methyltransferases in this sequence context. By using GCH (H = A, T, or C) cytosines to track nucleosome occupancy and HCG for endogenous DNA methylation, this technique could be adjusted to provide a simultaneous readout of nucleosome positioning and DNA methylation from the same DNA molecule across the human genome (Kelly et al., 2012). At gene promoters, nucleosome occupancy and DNA methylation patterns were consistent with transcription potential. The active promoters showed an unmethylated profile with a defined nucleosome free region. The inactive promoters were unmethylated as well but occupied by nucleosomes, whereas silent promoters were both methylated and fully nucleosomal. These findings demonstrate that only nucleosome positioning, or DNA methylation state of promoters as exclusive factors cannot predict the transcriptional state of a gene (Kelly et al., 2012).

In more recent years, third generation sequencing platforms including the Single-Molecule Real-Time sequencing (SMRT-seq) and Nanopore sequencing emerged (Clarke et al., 2009; Eid et al., 2009). For both techniques, sequencing library preparation does not include an amplification step, therefore enabling single-molecule sequencing. Importantly, these methods allow direct detection of modified bases in natural DNA samples without bisulfite conversion. In addition, single read lengths can reach the megabase range, giving the opportunity to accurately assemble and define repetitive elements and epigenetic modifications on variable chromatin stages and domains (Jain et al., 2016; Ardui et al., 2018).

**FIGURE 3:**
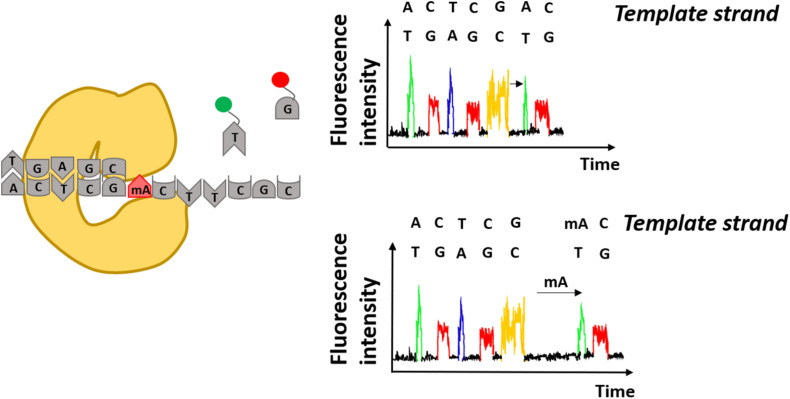
Detection of modified adenines using Single-Molecule Real Time sequencing. Specific fluorescence underlies incorporation of modified nucleotides by DNA polymerase. Differences in arrival times of the same nucleotides e.g., thymidine (T) indicate presence of modified nucleotides on the DNA template strand e.g., methylated adenine (mA).

The SMRT-seq approach by PacBio is based on fluorescently labeled nucleotides that are incorporated by DNA polymerase into the complementary DNA strand. The arrival times and durations of the individual fluorescent nucleotides provides information on the polymerase kinetics and the direct detection of covalently modified nucleotides including N6-methyladenine and 5-hydroxymethylcytosine (Figure 3). Each modification shows a unique kinetic signature and therefore allows to distinguish between them on the same DNA molecule (Flusberg et al., 2010). In the beginning, the accurate discrimination between a methylated and non-methylated base necessitated many controls and optimization steps. For that reason, a SMRT-BS method was reported combining bisulfite conversion with third-generation single-molecule real-time (SMRT) sequencing. The conceptual idea was to use the bisulfite conversion for accurate detection of CpG methylation on 1.5 kb regions and in parallel take advantage of SMRT sequencing with minimal clonal PCR artifacts (Yang et al., 2015).

Recently, using SMRT-seq only, two techniques named Fiber-seq and Single-Molecule-Adenine Methylated Oligonucleosome Sequencing Assay (SAMOSA) were developed using an unspecific N6-Methyladenine (m6A) DNA-methyltransferase for nucleosome detection in higher resolution (Abdulhay et al., 2020; Stergachis et al., 2020). Eukaryotic genomes are devoid of m6A but at the same time, adenines are present on double stranded DNA with an average rate of one every two DNA bases, providing unprecedented coverage of methylation sites and therefore higher resolution compared to CpG methylation.

Using this methodology, Fiber-seq could determine the primary architecture of single chromatin fibers at basepair resolution (Stergachis et al., 2020). Firstly, *Drosophila melanogaster* S2 cells were used to prove that DNA methyltranferases are unable to modify DNA wrapped in NCPs. The aim was to investigate whether the presence or absence of nucleosomes is the exclusive and most essential factor for the actuation of the regulatory elements or if the possible chromatin stages are more diverse and complex due to intermediate configurations. The results suggest that co-actuation exist on tightly clustered elements, meaning that high DNA accessibility of regulatory sequences at a given distal element can also increase accessibility of neighboring elements in a distance-dependent manner. Moreover, the obtained data demonstrate that DNA sequence alone might not define nucleosome positioning but it is mostly dependent on the actuation of regulatory DNA, since well-positioned nucleosomes were preferentially derived from fibers in which the regulatory element is in an actuated state (Stergachis et al., 2020). Similarly, SAMOSA was first applied as a proof of concept on *in vitro* assembled chromatin confirming its ability to track nucleosome positioning. As a next step, oligonucleosomes of K562 cells were used showing impartial nucleosome mapping on both euchromatin and heterochromatin regions (Abdulhay et al., 2020). Surprisingly, this study revealed elevated heterogeneity in both actively transcribed euchromatin and constitutive heterochromatin regions, the latter being typically viewed as a conformationally more static epigenomic domain.

**FIGURE 4:**
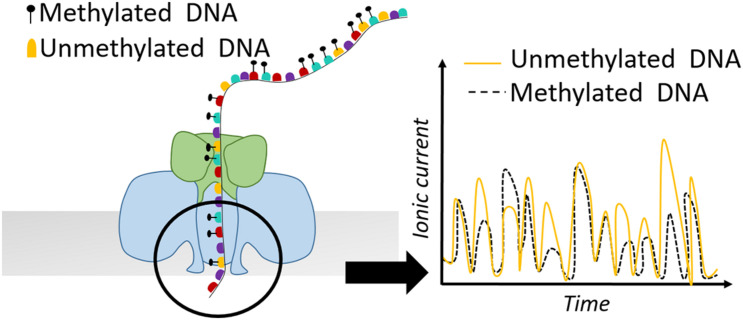
Schematic representation of a nanopore detecting ionic current of a DNA sequence. Methylated bases show different electric current signal compared to unmethylated residues.

Apart from SMRT-seq, nanopore sequencing provided by Oxford Nanopore Technologies (ONT) comprises another powerful tool for defining nucleosome positioning and chromatin accessibility. The idea of nanopore sequencing was developed by the observation that the different ionic current of DNA bases passing through a biological pore could allow discrimination of the different DNA bases (Kasianowicz et al., 1996). Apart from the canonical four DNA bases, different ionic current is also observed for methylated vs. non-methylated bases (Figure 4). In 2019, this property was exploited by **Me**thyltransferase treatment at GpC sites (by M.CviPI) on haploid *Saccharomyces cerevisiae* followed by **S**ingle-**M**olecule **L**ong-**R**ead sequencing (MeSMLR-seq) to probe long-range chromatin accessibility and nucleosome mapping at single DNA molecules (Wang Y. et al., 2019). The authors focused their analysis on transcription start sites (TSSs) and showed that silent genes show larger heterogeneity in nucleosome positioning compared to transcriptionally active genes. Additionally, MeSMLR-seq reads fully capture the coupled differences of chromatin accessibility of two genes in close neighborhood, providing the ability to uncover the heterogeneity not only within a cell population, but also at adjacent regions within the same molecule. Although bulk nucleosome mapping methods of the CLN2 promoter showed significant accessibility, it remained unknown if the open promoter conformation is uniform in the sense that the length of the accessible region among CLN2 promoters is identical. MeSMLR-seq revealed three distinct profiles of accessibility on CLN2 promoters: closed, narrowly opened and widely opened. This precise and accurate quantitative analysis expanded our current view on the different chromatin states and their heterogeneity at promoter elements (Wang Y. et al., 2019).

**S**ingle-**M**olecule long-read **A**ccessible **C**hromatin mapping sequencing (SMAC-seq) is another recently developed approach sharing the same concept with previous studies, but in parallel providing much higher resolution of the nucleosome landscape due to the simultaneous use of three different methyltransferases M.CviPI (GpC-5mC), M.SssI (CpG-5mC), and EcoGII (A at any sequence context-m6A) (Shipony et al., 2020). Given that this analysis has not been performed before, a combination of three different bioinformatic tools to detect methylation was necessary. Albacore was applied for the raw base calling, Tombo for m6A methylation detection, and Nanopolish for detection of CpG and GpC methylation. The findings of this study confirmed well-positioned nucleosomal patterns on yeast centromeric DNA in agreement with previous data. Moreover, it was shown that SMAC-seq is able to reveal binary chromatin states on rDNA, where approximately 25% of the individual molecules present full accessibility of the transcribed 35S region compared to the non-transcribed intergenic sequence (Shipony et al., 2020).

Taking the advantage of long read sequencing, ONT was also applied to four different human cell lines revealing the epigenetic status of single chromatin molecules based on endogenous CpG methylation and the chromatin accessibility measured by exogenous GpC methylation labeling (Lee et al., 2020). As a proof of concept, it was observed that regions closer to TSS exhibit higher CpG methylation status and lower GpC accessibility when H3K27me3 marks are present. However, after repression of H3K27me3, TSS promoter regions showed lower CpG methylation level and higher GpC accessibility. Thus, combining the two features- methylation and accessibility- and focusing on the same single reads around TSS could reveal characteristic epigenetic profiles, that are highly correlated with their corresponding expression profile (Lee et al., 2020).

## Single-Molecule Approaches to Monitor Histone PTMs and Effector Proteins

Besides the positioning of nucleosomes allowing or denying access to regulatory elements in the genome, the presence or absence of specific histone PTMs, histone variants, or other chromatin factors play pivotal roles for the functional state of the associated DNA. Ideally, one would need to measure multiple histone modifications at defined genomic loci and follow their dynamics live over time in single cells. Although such combinatorial in-depth analysis of histone PTMs still needs to be established at the single cell level, recent advances in imaging and sequencing techniques have started to uncover the dynamics of distinct histone modifications in various ways at the single cell level.

### Imaging Techniques

Imaging techniques emerge as powerful tools to detect histone PTMs at single cell and even single-molecule resolution. For example, High-throughput Histone Mapping (HiHiMap) allowed rapid and iterative quantification of histone PTMs as well as total histone levels in single cells throughout the cell cycle. Several histone modifications showed differences between normal and cancer cells, suggesting a potential role in oncogenic transformation (Zane et al., 2017). Another multicolor immunofluorescence technique used directly labeled modification-specific antibodies and profiled the dynamics of up to four histone modifications during the cell cycle (Hayashi-Takanaka et al., 2020). As epigenetic instability is a hallmark of cancer cells and many other human diseases, these high-throughput microscopy methods to quantify epigenetic marks also promise great potential to detect rare subpopulation of cells in patient samples and may inform on improved therapeutic strategies.

Förster resonance energy transfer (FRET) is a well-established method for studying structural and dynamic changes of nucleosomes at both the ensemble and the single-molecule level including PTMs (Simon et al., 2011) and presence of histone variants (Park et al., 2004; Hoch et al., 2007). These studies led to important insights, for example that mononucleosomes unwrap in a time window of ∼250 ms but rewrap more rapidly in 10–50 ms (Li et al., 2005). In addition, FRET based job plot experiments could reveal the stoichiometry of the trimeric CAF-1 histone chaperone leading to mechanistic insights how CAF-1 can deposit an (H3-H4)_2_ tetramer on substrate DNA in the first step of nucleosome assembly (Mattiroli et al., 2018; Sauer et al., 2018). More recently, single-molecule FRET (smFRET) of reconstituted, site-specifically labeled chromatin fibers revealed that NPCs undergo stacking interactions, which turnover on the micro- to millisecond timescale. Interestingly, binding of the heterochromatin protein HP1α transiently stabilizes stacked nucleosomes, resulting in a more compact albeit dynamic chromatin state (Kilic et al., 2018). In another smFRET study, the effect of histone acetylation and phosphorylation on nucleosome dynamics was analyzed (Brehove et al., 2015). Tyrosine 41 and threonine 45 phosphorylation of the H3 nucleosomal core resulted in enhanced DNA accessibility, similar to lysine 56 acetylation in the same core histone region. Remarkably, simultaneous phosphorylation and acetylation showed an additive effect and increased DNA accessibility more than 10-fold. These studies demonstrate the great potential of single-molecule fluorescence techniques to visualize nucleosome dynamics depending on histone PTMs and the binding of effector proteins. The vast amount of identified histone PTMs and other effector proteins await more single-molecule studies and promise great advance to our understanding of nucleosome structure and dynamics. However, interpretation of fluorescence microscopy data must always take into account the photophysical properties of the fluorophores regarding photostability, size, redox properties, and several other factors (Ha and Tinnefeld, 2012).

Force spectroscopy methods overcome these limitations as they detect the light scattered by micron-size particles. Thus, atomic force microscopy (AFM) has been extensively used to analyze chromatin at multiple levels, ranging from large chromatin fibers down to NCPs (Kalle and Strappe, 2012). In AFM, interactions between atoms of the tip and atoms of the sample leads to deflection that can be converted into topographic images of the sample (Zlatanova and van Holde, 2006). For example, high-speed time-lapse AFM demonstrated that nucleosomes undergo heterogenous and spontaneous disassembly and sliding events at the timescale of milliseconds, highlighting an important role of electrostatic interactions in chromatin dynamics (Miyagi et al., 2011). Optical and magnetic tweezers are two force manipulation methods that have been used to investigate nucleosome dynamics, histone DNA interactions as well as higher-order structural properties of chromatin (Killian et al., 2012). For example, magnetic tweezers experiments revealed that certain histone PTMs in vicinity to the dyad axis decrease the stability and promote eviction of nucleosomes (Simon et al., 2007). In an independent approach, single native chromatin molecules were subjected to nanochannels allowing the simultaneous high-throughput detection of fluorescent signatures of both the DNA and histone proteins within the chromatin. This single-chromatin analysis at the nanoscale (SCAN) method was used to determine the relationship of H3K9me3, H3K27me3, and CpG methylation in normal and cancer cells (Cipriany et al., 2010). Further studies employing such nano-platforms (Li et al., 2020) have sufficient throughput and high potential to provide a comprehensive picture of chromatin structure and may therefore also provide translational insights, such as cancer cell plasticity or the development of chemoresistance.

### Sequencing Techniques

The development of single cell ChIP-seq (scChIP-seq) technology provides histone modification detection at the genome-wide, single-cell level (Rotem et al., 2015). A droplet microfluidics-based procedure is necessary to limit possible sonication and sequencing library preparation problems. Although the low number (less than a thousand) reads per cell is still a significant challenge hampering broader applications, the authors reported H3K4me2 and H3K4me3 patterns in several cell lines, such as mouse ES cells, embryonic fibroblasts and hematopoietic progenitors that correlated well with bulk ChIP-Seq data. In addition, this paved the way for three new immunoprecipitation-free epigenomic profiling methods relying on *in situ* (inside nuclei) reactions. Single cell chromatin integration labeling followed by sequencing (scChIL-seq) (Harada et al., 2019) and the single cell cleavage under targets and tagmentation (scCUT&Tag) (Kaya-Okur et al., 2019) methods use specific antibodies against histone modifications and integrate a sequencing tag into double stranded DNA *via* transposase. This method was recently adapted to scalable nanowell and droplet-based single-cell platforms to profile polycomb group (PcG) silenced regions marked by H3K27me3 in tissue culture as well as patient samples (Wu et al., 2021). Similarly, combinatorial barcoding and targeted chromatin release (COBATCH) uses the enrichment of a Protein A-Tn5 transposase fusion protein to genomic regions by specific antibodies and produced for H3K27 acetylation marks ∼12,000 unique non-duplicated reads per cell (Wang Q. et al., 2019). The successful implementation and further developments of the Cleavage Under Targets & Release Using Nuclease (CUT&RUN) method by the Henikoff lab have now clearly revolutionized chromatin profiling methods as the use of MNase avoids sonication, material loss and background issues and thus overcomes many of the drawbacks of conventional and widely used chromatin immunoprecipitation (ChIP) methods (Meers et al., 2019). CUT&RUN-based single-cell chromatin immunocleavage sequencing (scChIC-Seq) uses an MNase-conjugated antibody which cleaves the non-nucleosomal DNA regions (Ku et al., 2019). Together, these latest developments of CUT&RUN-based methods toward single-cell applications promise great advances in epigenome profiling and insights into the heterogeneity of epigenetic marks even at the single cell resolution.

## Single-Molecule Approaches to Monitor Transcription

The different steps of the RNA Polymerase II (RNAP) transcription cycle have been studied in great detail by a combination of structural, biochemical and single-molecule approaches. Eukaryotic transcription is initiated by the binding of RNAP together with general transcription factors (GTF) onto the promoter region, the double stranded DNA is melted, and the transcription bubble is generated. When RNAP and GTF—TFIIA, TFIIB, TFIID, TFIIE, TFIIF, and TFIIH—assemble at promoters, they form a pre-initiation complex (PIC) to direct accurate transcription initiation. Although many of these key factors have been identified using bulk methods (Hofmann et al., 2004), important questions regarding the dynamic aspects or the mechanism of DNA opening and transcription start site (TSS) scanning required single-molecule approaches to provide sufficient resolution and to avoid population-averaging effects. Single-molecule approaches were also required to study transcription elongation, given that RNAP does not transcribe all DNA templates uniformly and at constant speeds (Kassavetis et al., 1978; Galburt et al., 2009). Instead, transcription elongation is interrupted by several pause and release states, such as promoter proximal escape (Muse et al., 2007), binding of transcription factors like TFIIS (Fish and Kane, 2002) nucleosomes (Kireeva et al., 2005) and association of splicing factors (Wachutka et al., 2019), which could not be revealed by population based methods.

### Force Manipulation Methods (Optical and Magnetic Tweezers)

**FIGURE 5:**
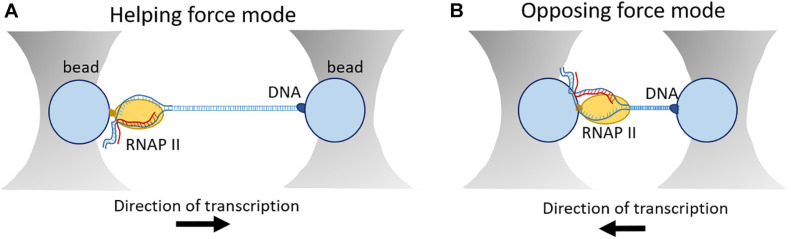
Outline of an optical tweezer set-up. **(A)** In the helping force mode, DNA is labeled upstream of RNAP leading to increased distance between the two beads and decreased force generation. **(B)** In the assisting force mode, DNA is labeled downstream of RNAP resulting in shortened distance between the two beads and increased force generation.

For this purpose, the development of optical tweezer technologies was instrumental towards a greater understanding of the dynamics of eukaryotic transcription on a single-molecule level. This technology was initiated in 1986 by the observation that interaction of a laser beam and small particles results in the formation of a three-dimensional trap with restoring forces on the order of piconewtons (Dziedzic et al., 1986). Regarding transcription, optical tweezers have been initially used for studies on elongation (Galburt et al., 2007, 2009; Larson et al., 2012; Schweikhard et al., 2014) and later they were also applied on transcription initiation (Fazal et al., 2015). In general, the experimental set-up is based on a “dumbbell” construction, consisting of two beads held in separate optical traps, connected by a segment of DNA. RNAP enzyme is attached to one bead and the opposite end of the DNA template is attached to another optical bead (Figure 5; Galburt et al., 2009; Fazal et al., 2015). T he force that is applied on the enzyme either helps or hampers transcription. Transcription elongation and nucleosome arrangement have been broadly investigated in the helping force mode. In this setup, one DNA molecule is labeled upstream of RNAP which results in increasing the distance between the two beads and therefore decreasing the generating force (Fitz et al., 2016). On the other hand, in the opposing mode, the tagged DNA is anchored downstream of RNAP. In this way, transcription reduces the distance between the two beads resulting in increased force that can be measured. For example, this setup was instrumental to determine the dynamics and frequency of pausing events of RNAP (Figure 5; Lisica and Grill, 2017).

Other applications of optical tweezer assays revealed that yeast RNAP II is unable to transcribe once the force of optical beads exceeds 8 pN. However, the addition of a single transcription factor (TFIIS) allows the polymerase to continue transcription against forces up to 20 pN (Galburt et al., 2007). An interesting interpretation of these data might be that the eukaryotic polymerase has evolved alongside with mechanical gene regulatory processes that control the ability of the polymerase to perform work (Galburt et al., 2009). In a large biochemical effort, a 32-protein, 1.5-megadalton yeast PIC could be assembled in an optical tweezer setup which gave important insights into the TFIIH-dependent DNA scanning mode that results in rapid DNA unwinding and in an extended transcription bubble formation with an average size of 85 bp (Fazal et al., 2015).

**FIGURE 6:**
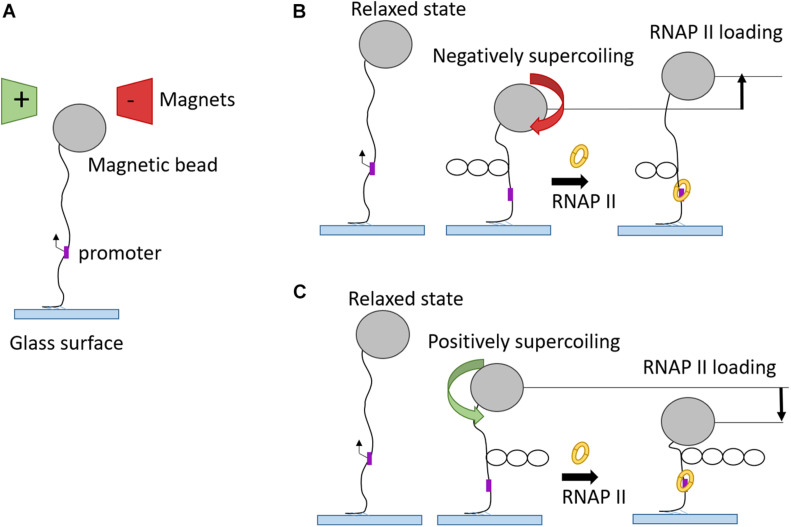
Magnetic tweezers used in transcription elongation studies. **(A)** Schematic representation of magnetic tweezers set-up in “relaxed” phase. **(B)** RNAP binding onto promoter of negatively supercoiled DNA substrate leads to unwinding of one turn and, therefore, to higher magnetic bead position. **(C)** RNAP binding onto promoter of positively supercoiled DNA substrate gives rise to one additional turn and, therefore, to a drop of magnetic bead position.

Complimentary to optical tweezer technology, magnetic tweezers were invented to allow single-molecule manipulation of paramagnetic beads using a magnetic field gradient. The first application of magnetic tweezers was in 1996 (Strick et al., 1996), who investigated the elasticity of supercoiled DNA. In a typical experiment, one side of a DNA molecule is attached to a paramagnetic bead and the opposite side is immobilized *via* streptavidin to a glass surface. Fixed magnets produce a magnetic field which pulls the bead in the direction of the field gradient. The magnets can be moved in a way to stretch or twist the single DNA molecule and these changes are recorded in real-time (Sarkar and Rybenkov, 2016). This approach offered great insight into the kinetics of promoter unwinding and clearance during transcription initiation (Revyakin et al., 2012). Positive or negative supercoiled DNA structure is formed by clockwise or anti-clockwise twists, respectively (Figure 6). After the binding to promoter DNA, RNAP unwinds the DNA template which leads to loss of one turn in the negatively supercoiled structure and to formation of one additional turn in the positively supercoiled DNA template. Magnetic tweezers were also used to test different models for PIC-mediated DNA opening and start site selection. Measuring the DNA bubble sizes generated by yeast PIC assembly showed that ATP hydrolysis by the subunit Ssl2 of TFIIH opens an initial 6 bp bubble that is extended by RNAP transcription to a larger 13 bp bubble (Tomko et al., 2017).

### Fluorescence-Based Methods for Transcription Initiation and Elongation

Another significant barrier to RNAP progression and therefore transcription elongation are nucleosomes, making their removal ahead of the transcription complex a necessity for productive transcription (Kulaeva et al., 2010). Optical tweezers have uncovered the crucial role of transcription factors TFIIH and TFIIF in avoiding transcription elongation pauses at nucleosomal barriers (Bintu et al., 2012; Ishibashi et al., 2014). However, one disadvantage of these methods could be the applied mechanical force, since it might lead to acceleration of transcription elongation or even to the eviction of individual nucleosomal barriers. A recently developed single-molecule FRET method could overcome these possible limitations and provided more accurate transcription kinetic measurements (Lee et al., 2019). The experimental set-up consists of a reconstituted nucleosome assembled on a DNA template containing yeast RNAP II that is attached to a polyethylenglycol coated glass surface *via* an Rpb1 C-terminal domain (CTD) antibody and a Protein A-Streptavidin complex. A FRET pair of labeled nucleotides that are in close proximity when wrapped in a nucleosomal core particle, but not in free DNA can then be used to detect the nucleosomal dynamics during transcription elongation (Lee et al., 2019). Using this system, it was found that the transcription elongation factors Spt4/5 are essential members of the elongation complex, bridging the “jaws” of RNAPII with nucleic acids in the transcription scaffold to allow nucleosomal transcription (Crickard et al., 2017).

The analysis of promoter-specific transcription initiation in a reconstituted human RNAP system was achieved at single-molecule resolution by fluorescence video microscopy (Revyakin et al., 2012). Fluorescently labeled templates with promoter DNA were immobilized on a polysiloxane-coated glass coverslip and incubated with NTPs and purified human transcription factors. The PIC assembly was monitored through specific interactions between transcription factors and DNA templates at sub-second time resolution and based on spatial colocalization of the two fluorescence signals recorded in two fluorescence optical channels (Revyakin et al., 2012). In this way, the authors could not only directly visualize interactions between transcription factors and DNA, but also track and directly count RNA production by individual promoters in real time.

## Single-Molecule Approaches to Monitor DNA Replication

The faithful transmission of epigenetic information undergoes a major challenge during DNA replication. Nucleosomes in front of the replication fork are disassembled and the corresponding parental histones with their PTMs need to be redeposited on the two daughter strands in order to maintain gene expression and cellular identities. Therefore, understanding how nascent chromatin is established, maintained or changed with the replication program is of paramount importance. Single-molecule approaches have provided valuable solutions to simultaneously observe replication fork proteins and DNA synthesis, furthering our understanding of replication fork dynamics.

### DNA Fiber Stretching and Molecular Combing to Detect Replication Fork Progression

Classic methods to detect cells undergoing DNA replication is based on measuring the incorporation of thymidine analogs like BrdU (5-bromo-2′-deoxyuridine) or EdU (5-ethynyl-2′-deoxyuridine) into newly synthesized DNA. The labeled DNA can then be quantified by flow cytometry or immunofluorescence (Pozarowski and Darzynkiewicz, 2004; Harris et al., 2018). Although these methods are widely used as a tool for quantitative analysis of replication profiles in a cell population, this approach is not able to resolve the heterogeneity and the complexity of individual replication forks undergoing DNA replication.

**FIGURE 7:**
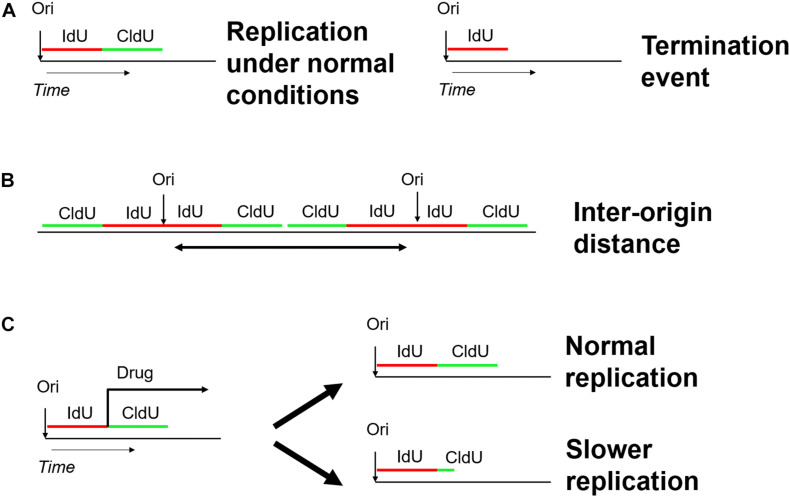
Schematic overview of the DNA fiber approach to analyze distinct replication events. Two subsequent pulses of the thymidine analogs IdU (5-iodo-2–deoxy-uridine) and CldU (5-chloro-2-deoxy-uridine) are used to label active DNA synthesis. **(A)** Study of replication initiation, elongation, and termination events. **(B)** Determining the distance between two neighboring replication origins. **(C)** Investigating the effect of genotoxic stress-inducing agents on replication progression.

Analysis of DNA fibers stretched either by DNA spreading or by molecular combing proved to be powerful tools to shed light into the dynamics of DNA and chromatin replication (Bensimon et al., 1994; Michalet, 1997; Tuduri et al., 2010). Both techniques share the same concept of two subsequent pulse labels with the different thymidine analogs iodo-deoxy-uridine IdU and chloro-deoxy-uridine CldU (Técher et al., 2013). The labeled DNA replication tracts are stained with antibodies directed against the halogenated nucleotides and the microscope slides with stretched DNA molecule are mounted for fluorescence microscopy. In contrast to DNA fiber spreading, molecular combing employs gentle lysis and slow stretching of DNA fibers on glass slides and enables the analysis of DNA replication on much larger DNA molecules up to 12 Mb in length (Kaykov et al., 2016). Each pulse (IdU/CldU) usually lasts 20–30 min and, consequently, an ongoing fork is represented as tracks of red and green labeled DNA (Nieminuszczy et al., 2016). The use of two different analogs gives the opportunity not only to detect the active replication forks in different chromosomes at a specific time-point (Czajkowsky et al., 2008), but also to extract additional parameters of DNA replication, including the replication fork speed, inter-origin distance, as well as initiation and termination events (Figure 7; Bialic et al., 2015; Nieminuszczy et al., 2016; Ray Chaudhuri et al., 2016; Vujanovic et al., 2017). The fork speed is calculated by the ratio between the fork length and the pulse time. Initiation events are represented as forks progressing symmetrically in opposite directions and termination events are merging forks.

Under normal conditions, this strategy has been used to quantify the progression of DNA replication forks in many organisms and cell culture systems, including chicken DT40 cells, Xenopus and mammalian cells (Blow et al., 2001; Schwab et al., 2010; Guilbaud et al., 2011). When combined with fluorescence *in situ* hybridization (FISH), this technique has even the potential to provide insights into the replication landscape at genomic loci of interest, such as Common Fragile Sites (Palumbo et al., 2010). DNA fiber analysis in different mutant cell lines could also reveal important interaction partners essential for replication initiation, e.g., the interaction of RECQ4 with MCM10 at replication origins (Kliszczak et al., 2015).

Under conditions of replication stress, fork speed is typically reduced as seen by DNA fiber analysis and this assay has been extensively used to assess the function of numerous proteins in replication fork progression and genome instability. A variation of the assay includes the treatment of cells with common replication inhibitors like hydroxyurea or aphidicolin, and monitor nucleotide resection following replication fork stalling by specific endonucleases. Successful implementation of this approach include the identification of the role of human RIF1 and Protein Phosphatase 1 in preventing over-degradation and accumulation of DNA breakage by replication stalling (Garzón et al., 2019; Mukherjee et al., 2019). Apart from the information regarding the overall slower rate of DNA replication or the fork degradation under specific conditions, the double labeling strategy is used to determine the frequency of fork stalling by measuring the ratio between two sister forks. In case of global slower rate of DNA polymerization, the sister forks would show the same length, as they are equally affected (ratio = 1). In case of fork stalling, one of the second tracts is typically shortened resulting in an asymmetric ratio< 1. This approach has been recently used to describe a mechanistic model of transcription-replication conflict resolution. It was shown that the restart of semi-conservative DNA replication is achieved by a fork cleavage mediated by the endonuclease MUS81/EME1 and religation cycle catalyzed by LIG4/XRCC4 (Chappidi et al., 2020).

Although DNA fiber assays are easy to implement in the laboratory and successfully used by many labs, one drawback is that the analysis of the molecules is labor-intense and low throughput. To improve this bottleneck, alternative methods use nanochannels for DNA stretching (Lacroix et al., 2016; De Carli et al., 2018). For example, an optical DNA mapping device (Bionano Genomics Irys) was repurposed to visualize DNA replication in Xenopus laevis egg extracts (De Carli et al., 2018). As a proof of principle, bacteriophage λ DNA was replicated in Xenopus egg extracts complemented with fluorescent deoxyuridine triphosphate (dUTP). Then, DNA was purified and labeled at specific restriction sites with a nicking endonuclease (NE) with another fluorescent nucleotide to allow optical alignment of the DNA molecules with the reference genome. Using this approach, the authors could confirm with high statistical significance that replication initiation in this system is not sequence-dependent (De Carli et al., 2018). Similar tools are currently developed in mammalian systems (Wang et al., 2020) and we envision that such methods may pave the way to DNA replication analysis at the genome-wide and single-molecule level.

### Psoralen Crosslinking-EM to Visualize Replication Forks

Psoralen crosslinking and EM have been used as complimentary approach to DNA fiber/combing to give structural insights into DNA replication process under normal and stressed conditions (Vindigni and Lopes, 2017; Zellweger and Lopes, 2018). Many different replication intermediates were captured under EM revealing the mechanistic role of many factors controlling replication. For example, absence of RAD53 kinase could lead to extensive single stranded gaps and to accumulation of Holiday junctions through fork reversal (Sogo, 2002). Moreover, DNA2-depletion and RECQ1-depletion are followed by increased fork reversal events demonstrating the important role of both factors in fork progression (Thangavel et al., 2015). To date, EM remains the only method allowing direct visualization of fork reversal, an important four-way junction that forms frequently upon replication perturbation (Neelsen and Lopes, 2015).

### Fluorescent and Force Manipulation Methods for DNA Replication Monitoring

*In vitro* reconstituted bacterial replisomes have been instrumental for the development of single-molecule techniques to visualize DNA replication. Using a rolling-circle amplification assay that flow-stretches the newly replicated DNA product on a functionalized glass coverslip (Tanner et al., 2008; Yao et al., 2009), important dynamics of individual components of the replication fork could be uncovered that were not possible by *in vitro* bulk biochemical experiments. For example, it was shown that T7 bacteriophage replisomes undergo frequent translocation in and out of replisomes in the process of DNA synthesis (Loparo et al., 2011), which could also be confirmed *in vivo* (Beattie et al., 2017). Another important aspect of the replisome concerns the coordination of leading and lagging-strand synthesis, as the continuous and discontinuous modes of replication at the two strands require strong coordination of the different sets of enzymatic reactions. Simultaneous Real-Time Imaging of Leading and Lagging Strand Synthesis showed that this coordination is regulated by both pausing of the leading-strand synthesis machinery and looping of the ssDNA (Duderstadt et al., 2016). Another single-molecule technique called DNA molecular curtains is based on the principle to align arrays of parallel nucleic acid strands in a flow chamber where the interaction with fluorescently labeled proteins can be observed in real time (Fazio et al., 2008). This high-throughput technique was used to study the transiently occurring intermediates of homologous recombination (HR) and provided for example crucial insights into the properties of individual Rad51 presynaptic complexes as one of the key intermediates in HR (Qi and Greene, 2016). With the successful biochemical reconstitution of eukaryotic *in vitro* DNA and chromatin replication in recent years (Yeeles et al., 2015; Kurat et al., 2017), we expect many more mechanistic insights also into eukaryotic replication fork and repair pathways using similar fluorescent single-molecule approaches.

Force manipulation methods are also suited for DNA replication studies. For example, using magnetic tweezers, the function of two different DNA polymerases (Sequenase and Klenow) was investigated on a single-strand and double-strand DNA (ssDNA)/(dsDNA) template. Sequenase is considered as a fast and Klenow as a slow polymerase (Maier et al., 2000). One end of the ssDNA was attached on a glass surface together with a primer allowing the binding of DNA polymerase and the other end was connected to a magnetic bead *via* streptavidin. The data shows that pausing of replication occurs at certain sites with both enzymes. However, there is no sequence specificity to these events, but the rate of replication was dependent on the stretching force applied to the DNA template. Low forces tend to increase the replication rate and forces stronger than 4 pN tend to decrease it. Pausing events were observed after a force greater than 20 pN is applied to the ssDNA. These events occur on multiple base pairs and follow the Arrhenius law. These results are consistent with other studies using optical tweezers set-up and demonstrate an increased probability of fork pausing events upon maximum tension of the DNA template (Wuite et al., 2000).

### Third Generation Sequencing to Detect DNA Replication

Nanopore sequencing technology was used for the first time to map DNA replication at a single-molecule level monitoring the incorporation of the thymidine analog BrdU (Hennion et al., 2018). After defining the characteristic signal current of BrdU on MinION nanopore device using an *in vitro* system, a basecalling algorithms was developed to recognize the BrdU-specific shift in electric current as a fifth DNA base. To train the analysis pipeline, a modified yeast strain was used that fully depends on exogenous addition of thymidine or BrdU for replication. This allowed accurate mapping of early replication origins in S phase (Hennion et al., 2018). The resolution and accuracy of this approach was further improved by D-Nascent (detecting nucleotide analog signal currents on extremely long nanopore traces) (Müller et al., 2019) using a software based on Hidden Markov Model to detect BrdU incorporation. Application of this technology in yeast and mammalian cells also revealed pausing events of replication forks at single-molecule resolution (Georgieva et al., 2020; Hennion, 2020).

## Conclusion and Perspectives

In this review, we summarized currently available approaches for chromatin analysis on a single-molecule level. The complex nature of chromatin with a myriad of dynamic interactions, large inter- and intramolecular heterogeneity and fast conformational changes among different states implies large stochasticity and variability on individual molecules that undergo replication, transcription and other nuclear processes. For this reason, single-molecule readouts are becoming increasingly important and at the same time feasible with the latest technological improvements in microscopy and third-generation sequencing.

These efforts have rewarded us with valuable insights into the dynamics of nucleosome positioning, transcription and replication dynamics that were not within our reach when only measuring bulk population of cells. Although many of these findings already proof to be consistent with bulk *in vivo* assays, we foresee that further development of live-cell imaging approaches will be needed to bridge the gap between the single-molecule *in vitro* assays and the behavior of the molecules in living cells.

Another aspect is that many of the single-molecule assays using nucleosomal templates rely on artificial, *in vitro* reconstituted mononucleosome or nucleosomal arrays that lack the majority of histone PTMs or *in vivo* positioning of nucleosomes. Purifying native chromatin from single loci would provide an interesting avenue for future single-molecule studies as such templates likely reflect better the *in vivo* situation (Hermans et al., 2017).

Third generation sequencing approaches might be the most promising technological development in recent years. Even though in the present review we mostly focus on applications in chromatin accessibility, their potential in other epigenetic profiling assays is enormous. Their revolutionized technology regarding the simple workflow, high throughput and long reads can also make them a useful diagnostic tool and with these new toolkits in hand, we expect many more important insights into basic and translational research in the next few years.

## Author Contributions

AC searched the literature, created the figures, and wrote and edited the manuscript. SH provided the guidance and wrote and edited the manuscript. Both authors contributed to the article and approved the submitted version.

## Conflict of Interest

The authors declare that the research was conducted in the absence of any commercial or financial relationships that could be construed as a potential conflict of interest.
