# Supplementary material for: Single-Molecule Techniques to Study Chromatin
Source: Front Cell Dev Biol. 2021 Jul 5;9:699771. doi: 10.3389/fcell.2021.699771 (PMC8287188; doi:10.3389/fcell.2021.699771)
Supplement: Supplementary file 1 [file Table_1.pdf]

Electron Microscopy/Atomic Force Microscopy

Principle	Name	Target/Process	Readout	Advantages	Disadvantages	References
**Intercalation of psoralen and UV-mediated crosslinking of double-stranded DNA**	Psoralen-EM	- Crosslinking of non-nucleosomal DNA<br>- EM under denaturing conditions to visualize nucleosome "bubbles"	Nucleosome positioning	- Direct visualization of nucleosomal footprint<br>- High resolution 5 to 10 bp	- Time-consuming and special equipment necessary<br>- No sequence/ locus information unless specific locus is highly enriched	Brown et al. 2013<br>Hamperl et al. 2014<br>Brown and Boeger 2014
		- Crosslinking of replicating chromatin	DNA replication intermediates	- Direct visualization of rare replication structures (ssDNA gaps, Holiday Junctions, Fork reversal)<br>- High resolution 5 to 10 bp		Sogo 2002<br>Thangavel et al. 2015
**Surface sensing using a nanoscale tip, deflections are converted in a topographic image**	Atomic Force Microscopy (AFM)	- Chromatin fibers and nucleosome particles	Nucleosome unfolding<br>Nucleosome sliding	- No special sample treatment/required<br>- High resolution in nanometer range	- Low throughput only single image about 150x150nm<br>- Low scanning time might cause thermal drift on the sample	Miyari et al. 2011

High-Content Imaging/Fluorescent Microscopy

Principle	Name	Target/Process	Readout	Advantages	Disadvantages	References
**High-content immunofluorescence microscopy against histones and histone PTMs**	HiHiMap<br><br>Multicolor fluorescence	- Single-cell readout/quantification across different cell cycle stages	Histone variants and Histone PTMs	- Multiplexed iterative analysis of multiple modifications<br>- High-Throughput<br>- Possibly useful for diagnostic applications	- Single-cell level but no single-molecule resolution<br>- Dependent on Fluorophore stability<br>- Application on fixed cells, no live-cell dynamics	Zane et al. 2017<br>Hayashi-Takanaka et al. 2020
**Incorporation of thymidine analogs into newly synthesized DNA**	DNA fiber stretching<br><br>DNA molecular combing	- DNA chromatin fibers with active replication forks	- Multiple readouts of DNA replication dynamics: Fork speed Inter-origin distance, Fork asymmetry, Termination	- DNA fiber assays easy to implement<br>- Monitoring a variety of replication events in the same assay<br>- Functional analysis possible how factors affect fork dynamics *in vivo*<br>- Megabase-sized fragments in DNA combing allows analysis of multiple replication events on same molecule	- Size of DNA fibers below typical inter-origin distance<br>- DNA combing requires specialized equipment<br>- No discrimination between the two newly replicating DNA strands<br>- No sequence/ locus information unless combined with experimentally challenging DNA FISH	Czajkowsky et al. 2008<br>Kaykov et al. 2016<br>Nieminuszczy et al. 2016<br>Vujanovic et al. 2017<br>Bialic et al. 2015<br>Kliszczak et al. 2015<br>Garzón et al. 2019<br>Chappidi et al. 2020
**Alignment of DNA molecules on a micro- and nanofabricated flowcell**	Molecular curtains	- DNA-protein interactions with fluorescently labeled proteins	- Nucleosome assembly	- Simultaneous imaging of hundreds or thousands of aligned molecules<br>- Detection of rare events with high statistical power	- Technically challenging due to potential uneven alignment of DNA or inefficient coverage<br>- Need individual optimizations due to nonspecific protein adsorption	Fazio et al. 2008<br>Qi and Greene 2018
			- DNA homologous recombination			
**Fluorescence Resonance Energy Transfer (FRET)**	FRET	- Distance-dependent transfer of energy from a donor fluorophore to an acceptor fluorophore	Transcription initiation and elongation	- Precise measurement of molecular distances<br>- Accurate transcription kinetics measurements without application of mechanical forces	- No information on which probe moves<br>- Free fluorophores can mask energy transfer<br>- FRET pair labelling needs to be bright with the donor completely saturated by acceptor	Lee et al. 2019<br>Crickard et al. 2017
	Single molecule FRET (smFRET)		Effect of histone PTMs and effector proteins on nucleosome dynamics			Kilic et al. 2018<br>Brehove et al. 2015

Force Spectroscopy Methods

Principle	Name	Target/Process	Readout	Advantages	Disadvantages	References
**Force generation upon interaction of laser beam with small particles**	Optical tweezers	- Two optical beads connected by single DNA segment –proteins are at	-Transcription elongation<br>-Nucleosome arrangement<br>- Dynamics and frequency of pausing events	- High force and temporal resolution measurements of 0.1pN and 10^{-12} sec<br>- Non-contact force in well-defined geometries	- Applied mechanical force could lead to artefactual behaviour<br>- Photodamage or thermal damage of the molecules	Fazal et al 2015<br>Galburt et al. 2009b<br>Galburt et al 2007
			-Histone-DNA interactions			Killian et al. 2012
**Force generation by magnetic field gradient**	Magnetic tweezers	- Stretch and twist of DNA molecule generated by magnetic field are recorded in real time<br>- Immobilized single DNA molecule connected on one side to paramagnetic bead	Promoter unwinding by RNAP and the role of PIC assembly in DNA opening	- High real-time resolution measurements<br>- Homogenous force field<br>- Simple application on DNA stretching<br>- Wide range of forces and lifetime of interactions from ms to hours	- Applied mechanical force could lead to misinterpretation<br>- Lower temporal and spatial resolution compared to optical tweezers	Revyakin et al. 2012<br><br>Tomko et al 2007
			The role of PTMs on nucleosome disassembly			Simon et al. 305 2007
			Pausing events on DNA replication			Maier et al. 2000<br>Wuite et al. 2000

Second and Third Generation DNA Sequencing applications

Principle	Name	Target/Process	Readout	Advantages	Disadvantages	References
**Single Cell Chromatin Immunoprecipitation**	scChIP-seq	- Indexed chromatin labelling from individual cells	Histone PTM analysis	- Chromatin landscape of single cell	- Microfluidic setup required for sonication/library preparation<br>- Low read depth<br>- Strong dependence on antibody quality against target	Rotem et al. 2015
**Cleave under targets and release under nuclease**	CUT&RUN	- Targeted chromatin regions are cleaved by MNase and released fragments directly sequenced	- Histone PTM detection<br>- Transcription factor<br>- Other chromatin factors	- Limited material loss as not relying on immunoprecipitation<br>- No need for sonication<br>- Suitable for low-input samples<br>- Low cost	- Adjustments needed for single-cell resolution<br>- Strong dependence on antibody quality against target	Meers et al. 2019
**Chromatin integration labeling via transposase**	scChIL-seq	- Antibody-DNA conjugate (ChIL probe) mediated transposase integration of sequences that allow T7 *in situ* transcription of genomic sequences in proximity to binding site of target protein or modification	- Histone PTM detection	- Immunoprecipitation free method<br>- Applicable to low input samples<br>- Specific detection of chromatin regions	- Single cell resolution only achieved for abundant histone PTM targets<br>- Bias of transposase reaction towards accessible chromatin regions<br>- Low read depth	Harada et al. 2019
	scCUT&Tag	- Tether Protein A-Transposase to primary chromatin target and integration coupled to microfluidics device	- Histone PTM detection			Kaya-Okur et al. 2019
**Barcoded and targeted chromatin release via transposase**	COBATCH	- Targeted chromatin regions are cleaved by protein A-Tn5 transposase fused to specific antibodies	- Histone PTM detection			Wang et al. 2019
**Bisulfite conversion of unmethylated cytosine to uracil**	Bisulfite-Sequencing (BS)	- Native chromatin treated with DNA methyltransferases<br>- PCR-amplified DNA fragments are sequenced directly or from single *E.coli* clones	- Nucleosome positioning<br>- Endogenous DNA methylation	- Accurate DNA methylation detection<br>- Easy to implement	- Low throughput and time- consuming workflow<br>- Short reads<br>- PCR amplification step can potentially lead to artifacts	Miranda et al. 2010<br><br>Kelly et al. 2012<br><br>Stadler et al. 2010
**PacBio Single Molecule Real Time Sequencing (SMRT)**	SMRT-BS	- Native chromatin fragments (~1.5kb) combined with bisulfite sequencing<br>- Incorporation of fluorescent nucleotides and analysis on optical nanostructures	- Open and closed chromatin regions<br>- Nucleosome positioning	- Sequencing in real time<br>- Enzymatic incorporation by DNA polymerase avoids signal loss over time and longer reads with low throughput (1,5kb)<br>- Limited artifacts by PCR and clonal selection<br>- Accurate methylation detection	- Clone selection (even though minimal compared to BS)<br>- Time-consuming workflow and limited throughput	Yang et al. 2015
	SAMOSA	- Chromatin fibers treated with DNA methyltransferases<br>- Discrimination of covalently modified and not modified nucleotides based on arrival times and the fluorescence duration	- Open and closed chromatin regions<br>- Nucleosome positioning	- Real time sequencing<br>- Usage of DNA polymerase avoids signal degradation over time<br>- Long reads with low throughput<br>- No PCR artifacts	- No standard bioinformatic pipeline available<br>- Optimization of methylation conditions<br>- Error-rate unclear when multiple methylation/modified bases present	Abdulhay et al. 2020
	Fiber-seq					Stergachis et al. 2020
**Oxford Nanopore Sequencing**	MeSMLR-seq	- Chromatin treated by DNA methyltransferases<br>- Discrimination between 4 canonical and modified DNA bases passing through a biological pore based on ionic current	- Open and closed chromatin regions<br>- Nucleosome positioning	- Real time analysis on personal computer<br>- Long reads up to megabase size<br>- No PCR artifacts<br>- Simple and rapid experimental set-up<br>- Direct detection of modified nucleotides without chemical modificatons	- No standard bioinformatic pipeline available<br>- Optimization of DNA methylation conditions essential<br>- Error-rate unclear when multiple methylation/modified bases present	Wang et al. 2019
	SMAC-seq					Shipony et al. 2020
	D-Nascent	- Detection of BrdU nucleotides based on ionic current as fifth DNA base to reveal reads from actively replicated DNA	- Fork progression<br>- Pausing events on DNA replication			Hennion et al. 2018<br>Müller et al. 2019<br>Hennion et al 2020<br>Georgieva et al. 2020
